# Supplementary material for: Theoretical Study of Hydrogen Production from Ammonia Borane Catalyzed by Metal and Non-Metal Diatom-Doped Cobalt Phosphide
Source: Molecules. 2022 Nov 24;27(23):8206. doi: 10.3390/molecules27238206 (PMC9741264; doi:10.3390/molecules27238206)
Supplement: Supplementary file 1 [file molecules-27-08206-s001.zip › molecules-2030480-supplementary.pdf]

molecules

Supplementary Materials

Theoretical Study of Hydrogen Production from Ammonia Borane Catalyzed by Metal and Non-metal Diatom-doped Cobalt Phosphide

Dong-Heng Li, Qiao-Mei Li, Shuang-Ling Qi, Hai-Chuan Qin, Xiao-Qin Liang and Laicai Li *

College of Chemistry and Material Science, Sichuan Normal University, Chengdu 610068, China
* Correspondence: lilcmail@163.com

Table of Contents

Additional Figures 2

Stable adsorption structures of and electron density maps**Error! Bookmark not defined.**

The process of NH_3BH_3 hydrogen production catalyzed by CoP..**Error! Bookmark not defined.**

The process of NH_3BH_3 hydrogen production catalyzed by $CoP_{Ni\text{-}N}$.. 3

The process of NH_3BH_3 hydrogen production catalyzed by $CoP_{Ga\text{-}N}$................................ 3

The process of NH_3BH_3 hydrogen production catalyzed by $CoP_{Ni\text{-}S}$.. 4

The energy level diagram of the reaction of five catalysts... 4

Density of states (DOS) and energy band structure 7

Additional Tables

Relevant structural parameters related to the hydrogen evolution reaction 8

Relevant energies and activation energies hydrogen evolution in reaction 10

Additional Figures

Stable adsorption structures and electron density maps

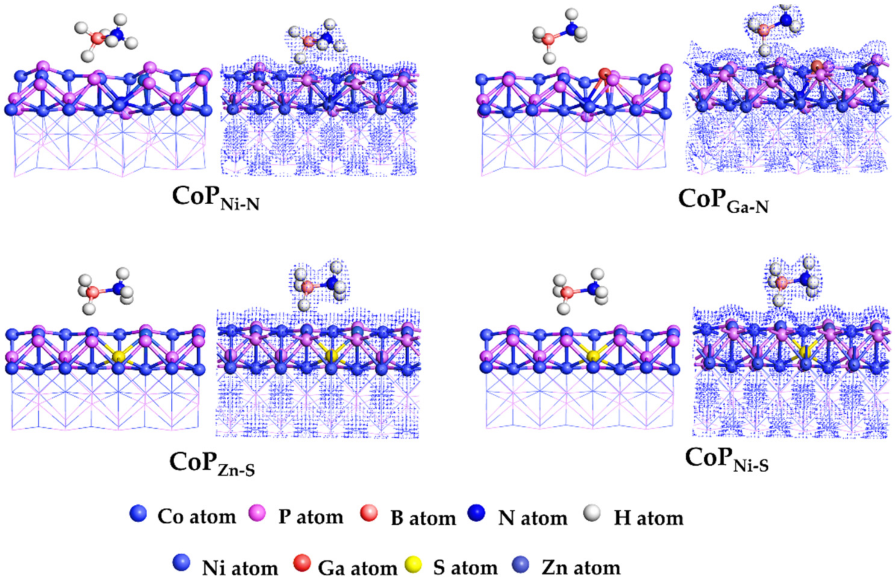

Figure S1. Stable structures and electron density maps of NH_3BH_3 absorbed on the surface of four different kinds of diatom-doped CoP catalysts (CoP_{Ni-N}, CoP_{Ga-N}, CoP_{Ni-S} and CoP_{Zn-S}).

The process of NH_3BH_3 hydrogen production catalyzed by CoP

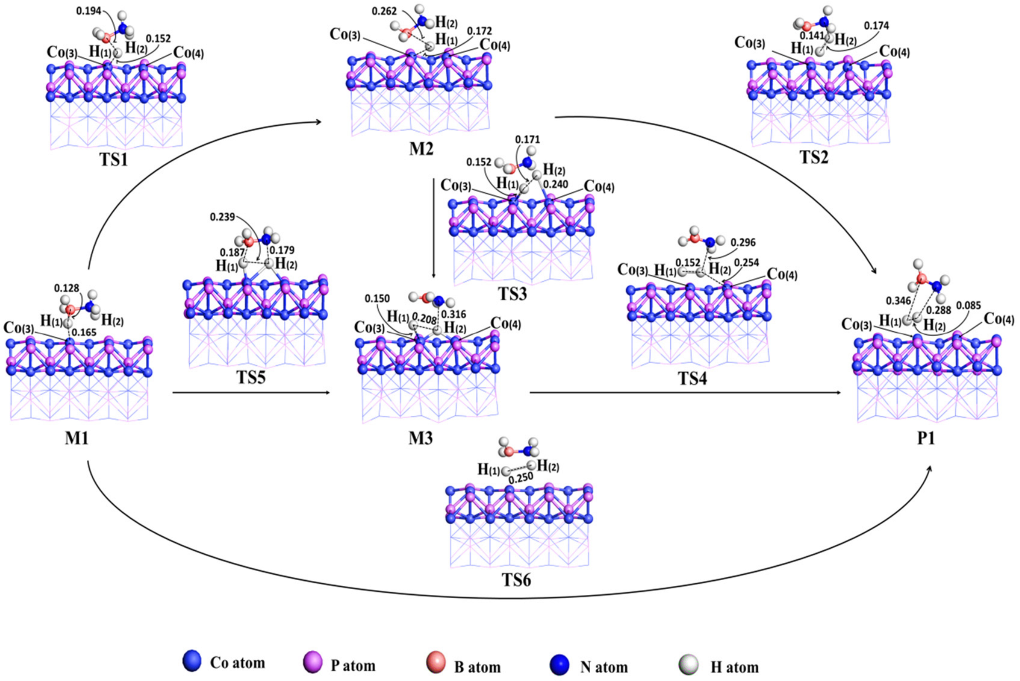

Figure S2. General flow chart of NH_3BH_3 hydrogen production reaction on CoP (101) surface.

The process of NH_3BH_3 hydrogen production catalyzed by $CoP_{Ni\text{-}N}$

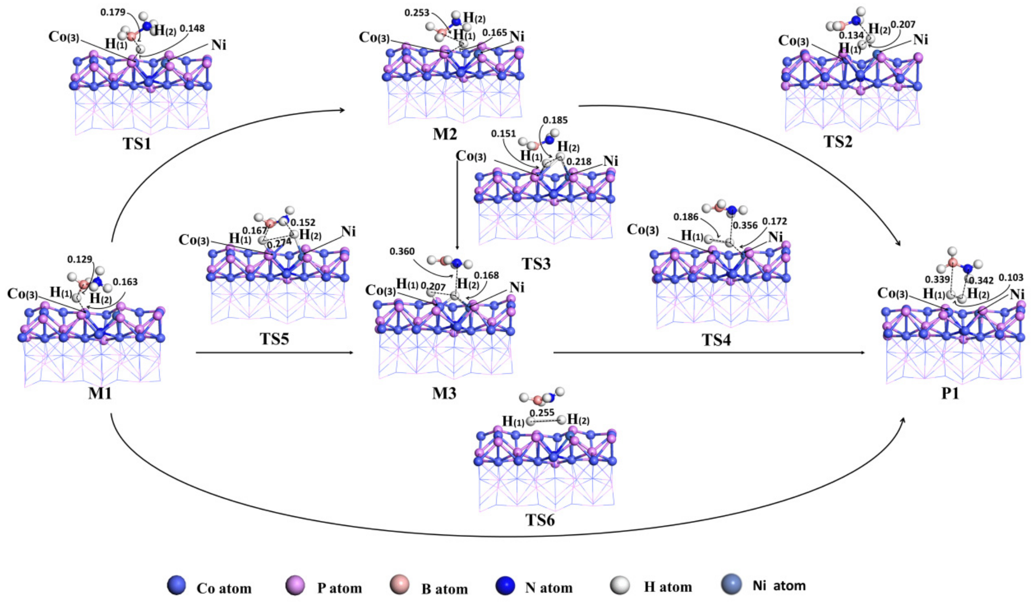

Figure S3. General flow chart of NH_3BH_3 hydrogen production reaction on $CoP_{Ni\text{-}N}$ (101) surface.

The process of NH_3BH_3 hydrogen production catalyzed by $CoP_{Ga\text{-}N}$

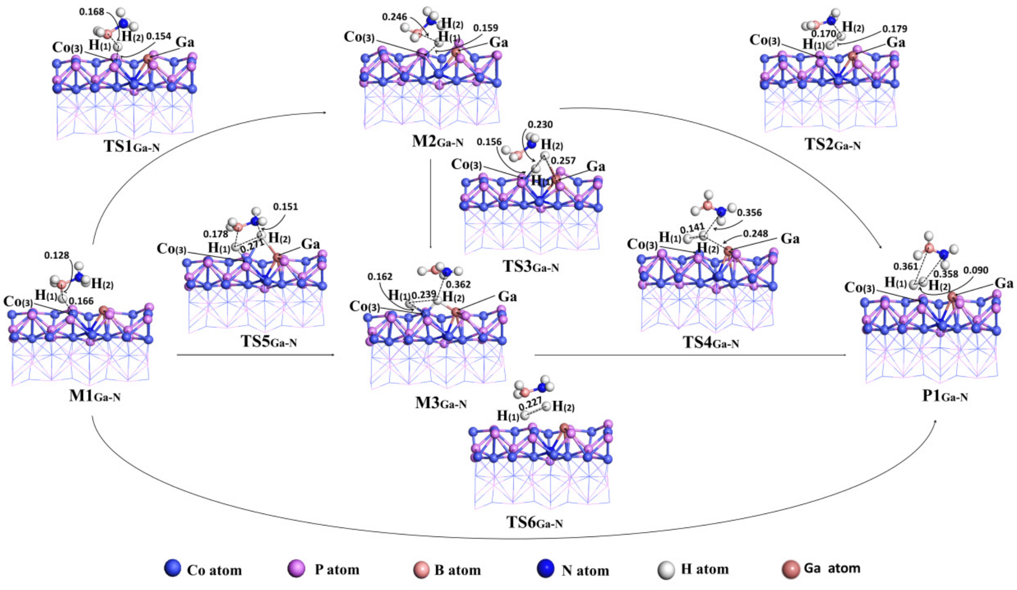

Figure S4. General flow chart of NH_3BH_3 hydrogen production reaction on $CoP_{Ga\text{-}N}$ (101) surface.

The process of NH_3BH_3 hydrogen production catalyzed by $CoP_{Ni\text{-}S}$

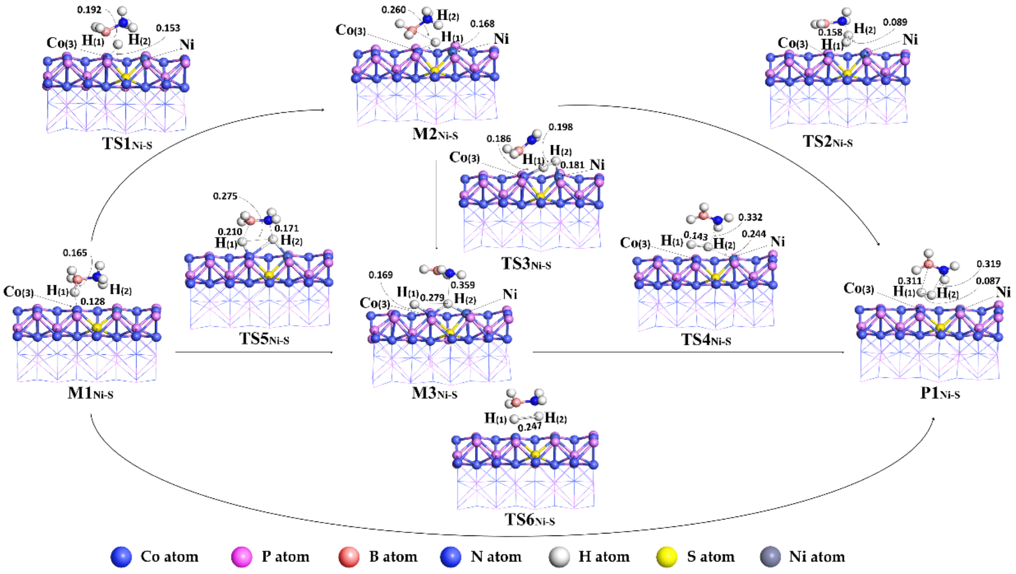

Figure S5. General flow chart of NH_3BH_3 hydrogen production reaction on $CoP_{Ga\text{-}N}$ (101) surface.

Energy level diagram of the reaction of five catalysts

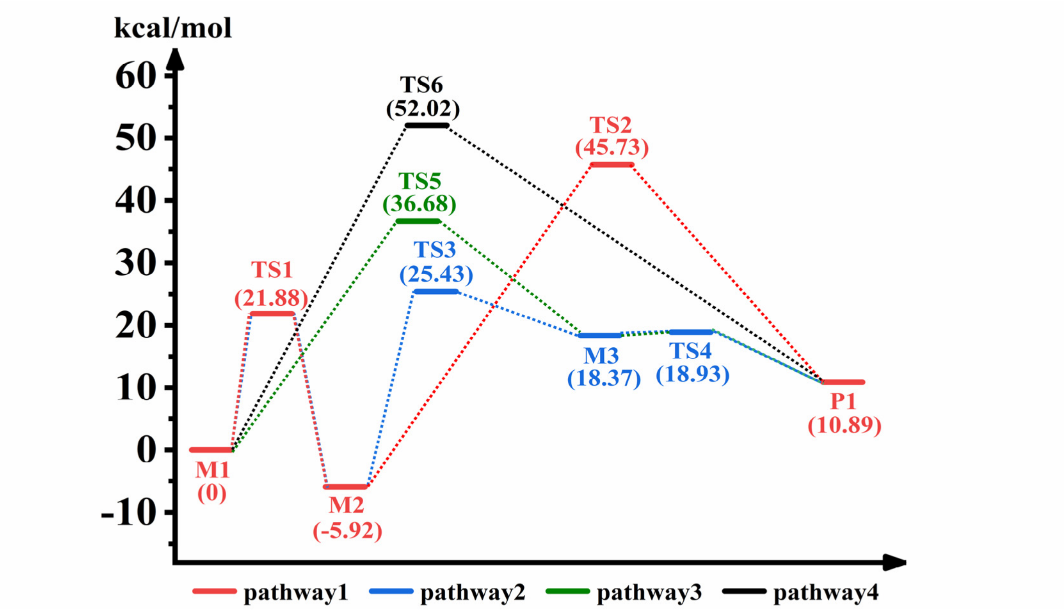

Figure S6. The energy profiles of NH_3BH_3 dehydrogenation reaction catalyzed by CoP.

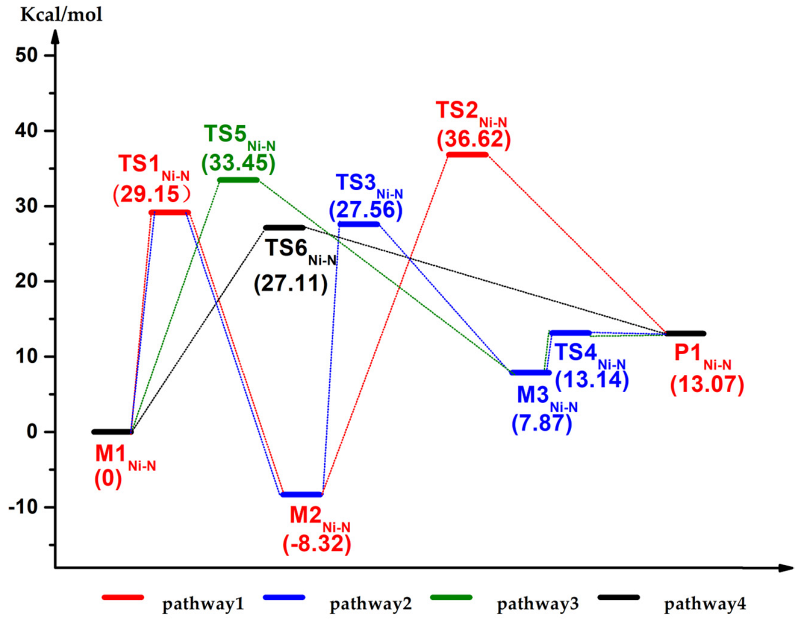

Figure S7. The energy profiles of NH_3BH_3 dehydrogenation reaction catalyzed by $CoP_{Ni\text{-}N}$.

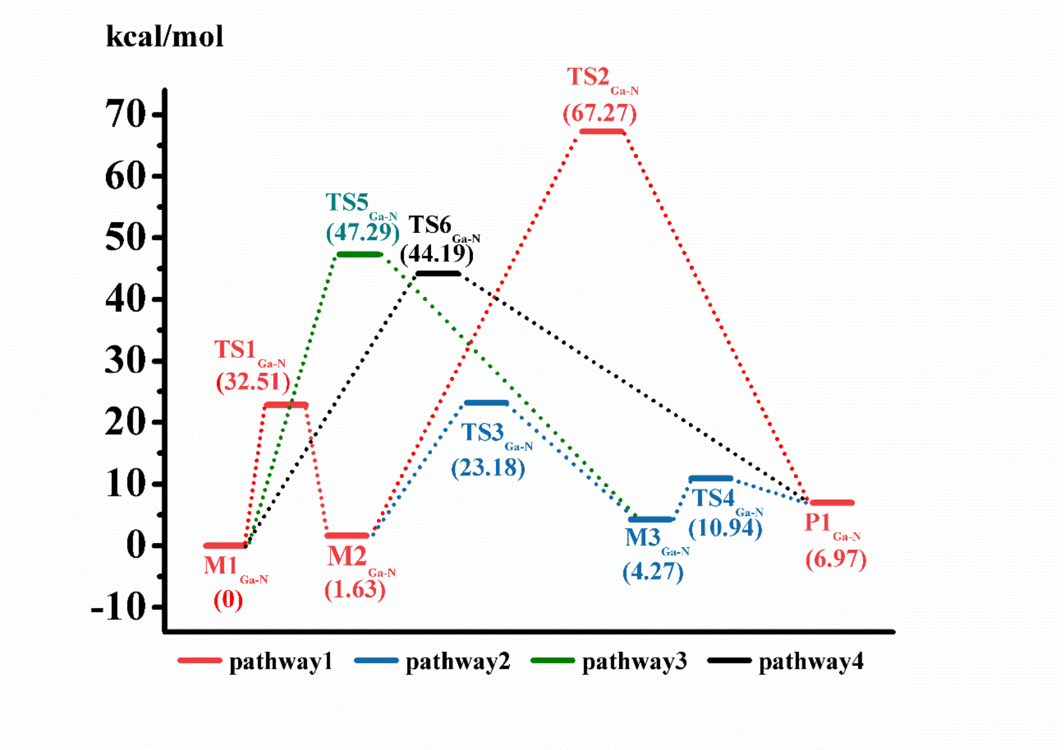

Figure S8. The energy profiles of NH_3BH_3 dehydrogenation reaction catalyzed by $CoP_{Ga\text{-}N}$.

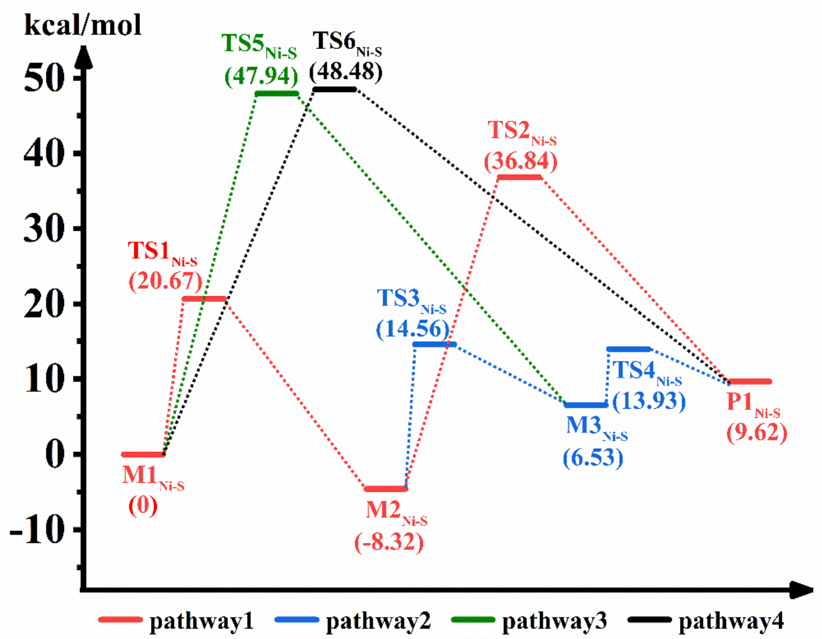

Figure S9. The energy profiles of NH3BH3 dehydrogenation reaction catalyzed by CoPGa-N.

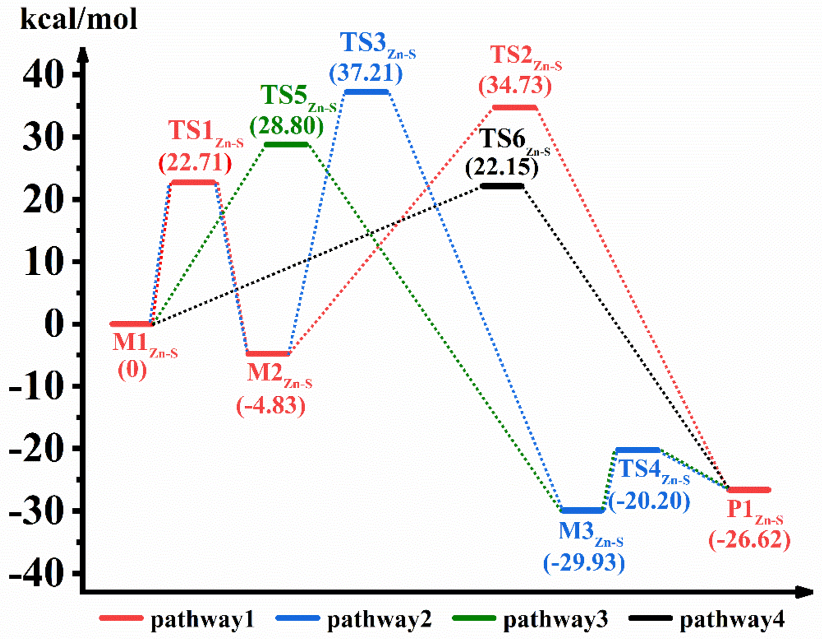

Figure S10. The energy profiles of NH3BH3 dehydrogenation reaction catalyzed by CoPGa-N.

Density of states (DOS) and energy band structure

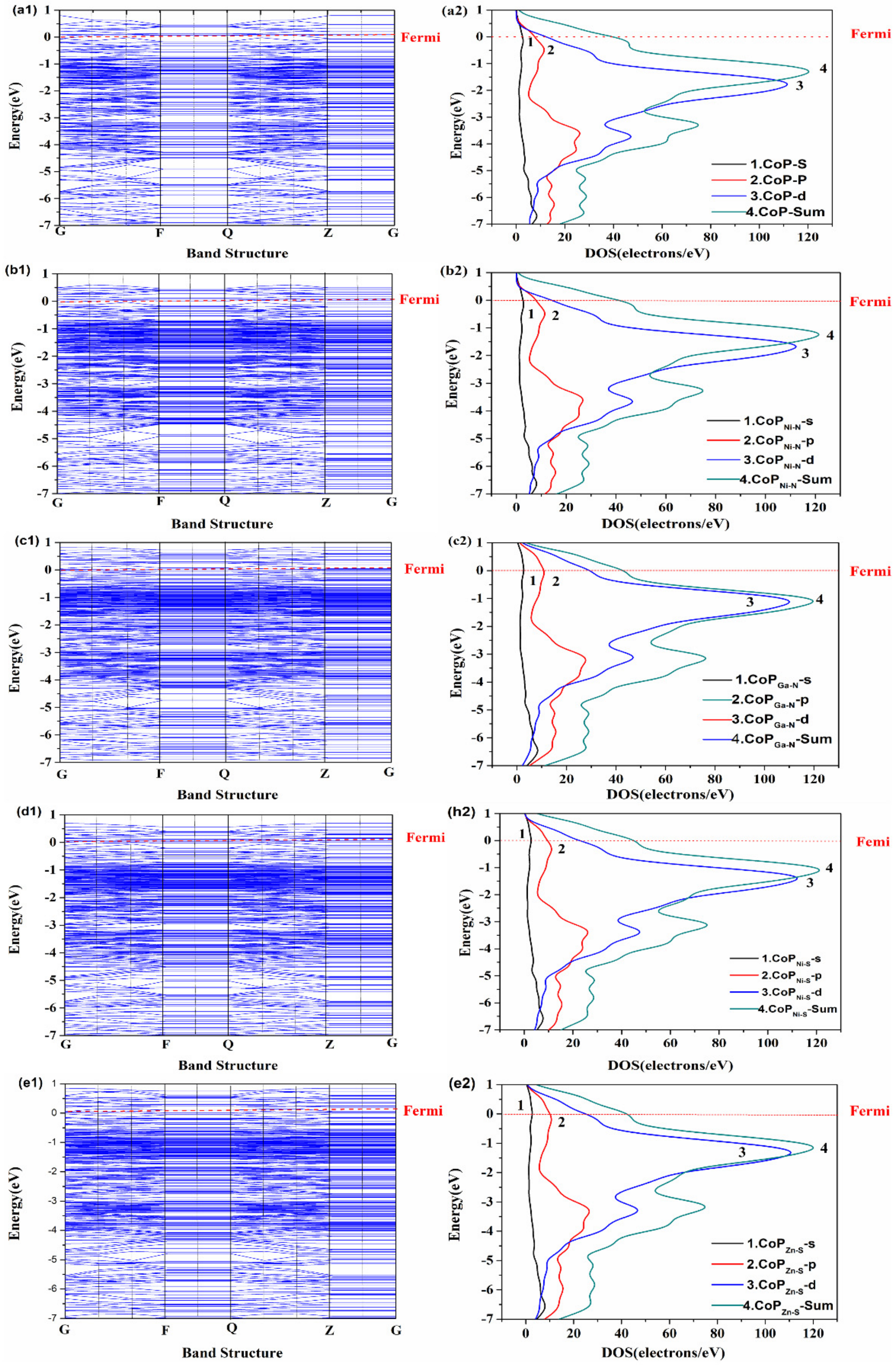

Figure S11. Schematic diagram of energy band structure(EBS) and density of states(DOS). (subscript 1 is EBS, subscript 2 is DOS, (a), (b), (c), (d), (e) refer to CoP, $CoP_{Ni\text{-}N}$, $CoP_{Ga\text{-}N}$, $CoP_{Ni\text{-}S}$、$CoP_{Zn\text{-}S}$ respectively).

Additional Tables.

Relevant structural parameters related to the hydrogen evolution reaction

Table S1. Bond lengths (nm) of various sites in the reaction of NH_3BH_3 catalyzed by CoP.

pathway		B-H(1)	Co(3)-H(1)	N-H(2)	Co(3)-H(2)	Ni-H(2)	H(1)-H(2)
I	M1	0.128	0.165	0.103	--	--	0.276
	TS1	0.194	0.152	0.103	--	--	0.204
	M2	0.262	0.172	0.103	--	--	0.193
	TS2	0.277	0.233	0.174	--	--	0.141
	P1	0.346	0.165	0.288	--	--	0.085
II	M1	--	0.165	0.103	0.345	0.303	0.276
	TS1	--	0.152	0.103	0.337	0.314	0.204
	M2	--	0.172	0.103	0.305	0.310	0.193
	TS3	--	0.152	0.184	0.285	0.240	0.171
	M3	--	0.150	0.316	0.168	0.173	0.208
	TS4	--	0.148	0.296	0.153	0.254	0.152
	P1	--	0.165	0.288	0.164	0.360	0.085
III	M1	0.128	0.165	0.103	0.345	0.303	0.276
	TS5	0.187	0.159	0.179	0.240	0.209	0.239
	M3	0.276	0.150	0.316	0.168	0.173	0.208
	TS4	0.306	0.148	0.296	0.153	0.254	0.152
	P1	0.346	0.165	0.288	0.164	0.360	0.085
IV	M1	0.128	--	0.103	--	--	0.276
	TS6	0.169	--	0.138	--	--	0.250
	P1	0.346	--	0.288	--	--	0.085

Table S2. Bond lengths (nm) of various sites in the reaction of NH_3BH_3 catalyzed by CoP_{Ni-N}.

		B-H(1)	Co(3)-H(1)	N-H(2)	Co(3)-H(2)	Ni-H(2)	H(1)-H(2)
I	$M1_{Ni-N}$	0.129	0.163	0.104	--	--	0.281
	$TS1_{Ni-N}$	0.179	0.148	0.102	--	--	0.195
	$M2_{Ni-N}$	0.253	0.165	0.104	--	--	0.207
	$TS2_{Ni-N}$	0.277	0.177	0.207	--	--	0.134
	$P1_{Ni-N}$	0.339	0.154	0.342	--	--	0.103
II	$M1_{Ni-N}$	--	0.163	0.104	0.306	0.260	0.281
	$TS1_{Ni-N}$	--	0.148	0.102	0.329	0.298	0.195
	$M2_{Ni-N}$	--	0.165	0.104	0.312	0.283	0.207
	$TS3_{Ni-N}$	--	0.151	0.202	0.270	0.218	0.185
	$M3_{Ni-N}$	--	0.149	0.360	0.162	0.168	0.207
	$TS4_{Ni-N}$	--	0.149	0.356	0.162	0.172	0.186
	$P1_{Ni-N}$	--	0.154	0.342	0.161	0.191	0.103
III	$M1_{Ni-N}$	0.129	0.163	0.104	0.306	0.260	0.281
	$TS5_{Ni-N}$	0.167	0.160	0.152	0.276	0.225	0.274
	$M3_{Ni-N}$	0.329	0.149	0.360	0.162	0.168	0.207
	$TS4_{Ni-N}$	0.327	0.149	0.356	0.162	0.172	0.186
	$P1_{Ni-N}$	0.339	0.154	0.342	0.161	0.191	0.103
IV	$M1_{Ni-N}$	0.129	--	0.104	--	--	0.281
	$TS6_{Ni-N}$	0.168	--	0.149	--	--	0.255
	$P1_{Ni-N}$	0.339	--	0.342	--	--	0.103

Table S3. Bond lengths (nm) of various sites in the reaction of NH_3BH_3 catalyzed by CoP_{Ga-N}.

pathway		B-H(1)	Co(3)-H(1)	N-H(2)	Co(3)-H(2)	Ni-H(2)	H(1)-H(2)
I	$M1_{Ni-S}$	0.128	0.165	0.104	--	--	0.276
	$TS1_{Ni-S}$	0.192	0.153	0.104	--	--	0.206
	$M2_{Ni-S}$	0.260	0.168	0.104	--	--	0.201
	$TS2_{Ni-S}$	0.273	0.187	0.158	--	--	0.089
	$P1_{Ni-S}$	0.311	0.162	0.319	--	--	0.087
II	$M1_{Ni-S}$	--	0.165	0.104	0.315	0.272	0.276
	$TS1_{Ni-S}$	--	0.153	0.104	0.327	0.282	0.206
	$M2_{Ni-S}$	--	0.168	0.104	0.306	0.281	0.201
	$TS3_{Ni-S}$	--	0.186	0.269	0.317	0.181	0.198
	$M3_{Ni-S}$	--	0.169	0.359	0.172	0.176	0.279
	$TS4_{Ni-S}$	--	0.149	0.332	0.154	0.244	0.143
	$P1_{Ni-S}$	--	0.162	0.319	0.164	0.271	0.087
III	$M1_{Ni-S}$	0.128	0.165	0.104	0.315	0.272	0.276
	$TS5_{Ni-S}$	0.210	0.151	0.171	0.256	0.203	0.275
	$M3_{Ni-S}$	0.385	0.169	0.359	0.172	0.176	0.279
	$TS4_{Ni-S}$	0.327	0.149	0.332	0.154	0.244	0.143
	$P1_{Ni-S}$	0.311	0.162	0.319	0.164	0.271	0.087
IV	$M1_{Ni-S}$	0.128	--	0.104	--	--	0.276
	$TS6_{Ni-S}$	0.163	--	0.247	--	--	0.143
	$P1_{Ni-S}$	0.311	--	0.319	--	--	0.087

Table S4. Bond lengths (nm) of various sites in the reaction of NH_3BH_3 catalyzed by CoP_{Ni-S}.

pathway		B-H(1)	Co(3)-H(1)	N-H(2)	Co(3)-H(2)	Ni-H(2)	H(1)-H(2)
I	$M1_{Ga-N}$	0.128	0.166	0.103	--	--	0.265
	$TS1_{Ga-N}$	0.168	0.154	0.104	--	--	0.222
	$M2_{Ga-N}$	0.246	0.159	0.105	--	--	0.245
	$TS2_{Ga-N}$	0.269	0.155	0.170	--	--	0.179
	$P1_{Ga-N}$	0.361	0.158	0.358	--	--	0.090
II	$M1_{Ga-N}$	--	0.166	0.103	0.304	0.296	0.265
	$TS1_{Ga-N}$	--	0.154	0.104	0.336	0.292	0.222
	$M2_{Ga-N}$	--	0.159	0.105	0.337	0.298	0.245
	$TS3_{Ga-N}$	--	0.156	0.152	0.317	0.257	0.230
	$M3_{Ga-N}$	--	0.162	0.362	0.158	0.190	0.239
	$TS4_{Ga-N}$	--	0.160	0.356	0.156	0.248	0.141
	$P1_{Ga-N}$	--	0.158	0.358	0.158	0.278	0.090
III	$M1_{Ga-N}$	0.128	0.166	0.103	0.304	0.296	0.265
	$TS5_{Ga-N}$	0.178	0.163	0.151	0.270	0.256	0.271
	$M3_{Ga-N}$	0.393	0.162	0.362	0.158	0.190	0.239
	$TS4_{Ga-N}$	0.369	0.160	0.356	0.156	0.248	0.141
	$P1_{Ga-N}$	0.361	0.158	0.358	0.158	0.278	0.090
IV	$M1_{Ga-N}$	0.128	--	0.103	--	--	0.265
	$TS6_{Ga-N}$	0.191	--	0.166	--	--	0.227
	$P1_{Ga-N}$	0.158	--	0.358	--	--	0.090

Relevant energies and activation energies hydrogen evolution in reaction

Table S5. The each position energies (E), relative energies (E_{rel}) and activation energies (E_a) of ammoborane reaction catalyzed by CoP.

pathway	compound	E_{rel}	E_a
		kcal/mol	kcal/mol
pathway I	M1	0.00	
	TS1	21.88	21.88
	M2	-5.92	

	TS2	45.73	51.65
	P1	10.89	
pathway II	M1	0.00	
	TS1	21.88	21.88
	M2	-5.92	
	TS3	25.43	31.35
	M3	18.37	
	TS4	18.93	0.57
	P1	10.89	
pathway III	M1	0.00	
	TS5	36.68	36.68
	M3	18.37	
	TS4	18.93	0.57
	P1	10.89	
pathway IV	M1	0.00	
	TS6	52.02	52.02
	P1	10.89	

Table S6. The each position energies (E), relative energies (E_{rel}) and activation energies (E_a) of ammoborane reaction catalyzed by $CoP_{Ni\text{-}N}$.

pathway	compound	E_{rel} kcal/mol	E_a kcal/mol
pathway I	$M1_{Ni\text{-}N}$	0.00	
	$TS1_{Ni\text{-}N}$	29.15	29.15
	$M2_{Ni\text{-}N}$	-8.25	
	$TS2_{Ni\text{-}N}$	36.62	44.87
	$P1_{Ni\text{-}N}$	13.07	
pathway II	$M1_{Ni\text{-}N}$	0.00	
	$TS1_{Ni\text{-}N}$	29.15	29.15
	$M2_{Ni\text{-}N}$	-8.25	
	$TS3_{Ni\text{-}N}$	27.56	35.81
	$M3_{Ni\text{-}N}$	7.87	
	$TS4_{Ni\text{-}N}$	13.14	5.27
	$P1_{Ni\text{-}N}$	13.07	
pathway III	$M1_{Ni\text{-}N}$	0.00	
	$TS5_{Ni\text{-}N}$	33.45	33.45
	$M3_{Ni\text{-}N}$	7.87	
	$TS4_{Ni\text{-}N}$	13.14	5.27
	$P1_{Ni\text{-}N}$	13.07	
pathway IV	$M1_{Ni\text{-}N}$	0.00	
	$TS6_{Ni\text{-}N}$	27.11	27.11
	$P1_{Ni\text{-}N}$	13.07	

Table S7. The each position energies (E), relative energies (E_{rel}) and activation energies (E_a) of ammoborane reaction catalyzed by $CoP_{Ga\text{-}N}$.

pathway	compound	E_{rel} kcal/mol	E_a kcal/mol
pathway I	$M1_{Ga\text{-}N}$	0.00	
	$TS1_{Ga\text{-}N}$	22.84	22.84
	$M2_{Ga\text{-}N}$	1.63	
	$TS2_{Ga\text{-}N}$	67.27	65.63
	$P1_{Ga\text{-}N}$	6.97	
pathway II	$M1_{Ga\text{-}N}$	0.00	
	$TS1_{Ga\text{-}N}$	22.84	22.84
	$M2_{Ga\text{-}N}$	1.63	
	$TS3_{Ga\text{-}N}$	23.18	23.18
	$M3_{Ga\text{-}N}$	4.27	
	$TS4_{Ga\text{-}N}$	10.94	6.67
	$P1_{Ga\text{-}N}$	6.97	
pathway III	$M1_{Ga\text{-}N}$	0.00	
	$TS5_{Ga\text{-}N}$	47.29	47.29
	$M3_{Ga\text{-}N}$	4.27	
	$TS4_{Ga\text{-}N}$	10.94	6.67
	$P1_{Ga\text{-}N}$	6.97	
pathway IV	$M1_{Ga\text{-}N}$	0.00	
	$TS6_{Ga\text{-}N}$	44.19	44.19
	$P1_{Ga\text{-}N}$	6.97	

Table S8. The each position energies (E), relative energies (E_{rel}) and activation energies (E_a) of ammoborane reaction catalyzed by $CoP_{Ni\text{-}S}$.

pathway	compound	E_{rel} kcal/mol	E_a kcal/mol
pathway I	$M1_{Ni\text{-}S}$	0.00	
	$TS1_{Ni\text{-}S}$	20.67	20.67
	$M2_{Ni\text{-}S}$	-4.58	
	$TS2_{Ni\text{-}S}$	36.84	45.16
	$P1_{Ni\text{-}S}$	9.62	
pathway II	$M1_{Ni\text{-}S}$	0.00	
	$TS1_{Ni\text{-}S}$	20.67	20.67
	$M2_{Ni\text{-}S}$	-4.58	
	$TS3_{Ni\text{-}S}$	14.56	19.14
	$M3_{Ni\text{-}S}$	6.53	
	$TS4_{Ni\text{-}S}$	13.93	7.40
	$P1_{Ni\text{-}S}$	9.62	
pathway III	$M1_{Ni\text{-}S}$	0.00	
	$TS5_{Ni\text{-}S}$	47.94	47.94
	$M3_{Ni\text{-}S}$	6.53	
	$TS4_{Ni\text{-}S}$	13.93	7.40
	$P1_{Ni\text{-}S}$	9.62	
pathway IV	$M1_{Ni\text{-}S}$	0.00	
	$TS6_{Ni\text{-}S}$	48.48	48.48
	$P1_{Ni\text{-}S}$	9.62	
